# The impact of Loop Electrosurgical Excision Procedure and cold-knife conization training model on the surgical skills and confidence level

**DOI:** 10.4274/tjod.galenos.2020.25675

**Published:** 2020-07-29

**Authors:** İlker Selçuk, Burak Ersak, Mutlu Umaroğlu, Şule Özel, Hakan Yalçın, Yusuf Üstün, Yaprak Engin-Üstün

**Affiliations:** 1 University of Health Sciences Turkey, Zekai Tahir Burak Woman’s Health Training and Research Hospital, Clinic of Gynecologic Oncology, Ankara, Turkey; 2 University of Health Sciences Turkey, Zekai Tahir Burak Woman’s Health Training and Research Hospital, Clinic of Gynecology, Ankara, Turkey; 3 Hacettepe University Faculty of Medicine, Department of Bio-Statistics, Ankara, Turkey; 4 University of Health Sciences Turkey, Ankara Training and Research Hospital, Clinic of Gynecology, Ankara, Turkey

**Keywords:** LEEP, conization, surgery, cervix, education

## Abstract

**Objective:**

The purpose of this study is to evaluate the impact of loop electrosurgical excision procedure (LEEP) and cold-knife conization (CKC) active training model on the surgical education and confidence levels of gynecologists.

**Materials and Methods:**

The LEEP and CKC hands-on training model consists of sausage, which is 2.5 cm in diameter, as cervix; plastic cup as vagina; foam rubber as posterior and anterior fornices; and cotton plate as the leukoplakia area. In total, 34 participants performed LEEP and CKC procedures on the training model under the guidance of mentors after theoretical lessons about the transformation zone, indications, and surgical techniques of LEEP and CKC. Afterward, a web-based survey was conducted to measure the effectiveness of this surgical model, and participants graded their learning and confidence levels on the same.

**Results:**

We evaluated the educational levels of the course, which were based on the basic surgical steps of LEEP and CKC procedures, and the confidence levels of the participants with regard to the previous practice or expertise of LEEP and CKC. Importantly, participants in each group had similar learning gains irrespective of previous practice or expertise. Despite a significantly higher pre-course confidence level of participants who had previously performed LEEP (p<0.001) and CKC (p<0.001) against their non-practitioner counterparts, the post-course confidence levels were similar in each group (p=0.127 and p=0.845, respectively). In both groups, the participants had increased mean confidence scores, which were statistically significant for participants who had not previously performed the procedures (p=0.003, LEEP and p=0.002, CKC, respectively).

**Conclusion:**

This surgical training model on LEEP and CKC can impart a better level of education in participants, irrespective of their previous expertise/practice.

**PRECIS:** The surgical training model on LEEP and cold knife conisation will be used to improve the surgical skills and confidence level of gynecologists.

## Introduction

Loop electrosurgical excision procedure (LEEP) and cold-knife conization (CKC) are well-known surgical operations that are applied in the treatment of cervical intraepithelial neoplasia (CIN) with similar success rates^(1)^. CIN is a precancerous lesion that is classified in three grades. The second- and third-grade (high grade) lesions can persist and progress further into invasive cervical cancer in the span of 10-20 years^(2)^. Additionally, after a colposcopic examination, LEEP or CKC will provide a complete histologic evaluation of the transformation zone both for the treatment of high grade CIN and diagnosis of a cervical malignity^(3)^.

LEEP is frequently performed in the gynecology practice, especially in office settings. Despite being a relatively easy procedure, the surgeon should be careful during the excision of cervical tissue in achieving minimal thermal artifact^(4)^. Nevertheless, the most common complications such as bleeding and infection are not as dangerous as the risk of future obstetric complications. CKC is also an excisional procedure, which is performed at the operation theater/room under general anesthesia mostly with an excessive tissue volume as compared to LEEP^(5)^. The risks of bleeding and other unfavorable obstetric outcomes, especially preterm labor, are slightly higher after CKC^(6)^.

As compared with CKC, LEEP is a simple procedure, which is performed at the office setting. Additionally, the need of experience and practice to complete the learning curve of LEEP is lesser than CKC^(7)^. During obstetrics and gynecology residency, many residents attend to LEEP and CKC operations. However, to gain the adequate experience, residents should perform these procedures independently; otherwise, there will be a lack of education. The lack of standardized surgical education will lead to many undesirable outcomes^(8)^. Many training models have been proposed so far to improve the surgical knowledge, skills, and confidence levels of a surgeon before he/she performs a live surgery^(9,10)^. This research focuses on the efficacy of a training model of LEEP and CKC, which mimics the surgical procedure and conditions.

## Materials and Methods

**Figure 1:**
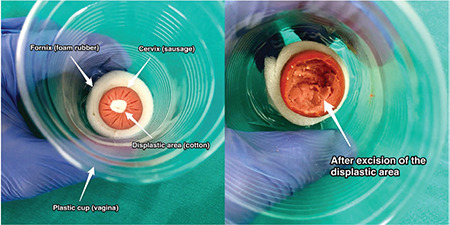
Loop Electrosurgical Excision Procedure and cold-knife conization hands-on training model

The purpose of this study is to measure the role of LEEP and CKC active training model in imparting education in gynecologists after they attended “LEEP and Cold-Knife Conization Hands-On Practice Workshop, 2018”, which was held at the University of Health Sciences Turkey, Zekai Tahir Burak Woman’s Health Training and Research Hospital, Ankara, Turkey. This one-day program was based on both theoretical and practice sessions. After the sessions, the responses of the participants were recorded on a web-based questionnaire. Importantly, after attending detailed didactic lectures about the transformation zone, the indications of excisional procedures for cervical pathologies, and the surgical techniques of LEEP and CKC, the participants watched the videos of LEEP and CKC procedures. The LEEP and CKC hands-on training model comprised sausage, 2.5 cm in diameter as cervix; plastic cup as vagina; foam rubber as posterior and anterior fornices; and cotton plate as leukoplakia area (Figure 1).

During the practice session, the senior surgeons in gynecology and gynecologic oncology practice first performed LEEP and CKC on the model and showed the procedure to 34 participants. The neutral electrode was positioned on the posterior part of the training model and then attached to the sausage to maintain the current of electrocautery. Different sizes of LEEP loops were used to excise the transformation zone entirely in one movement, neither slowly nor quickly. During CKC, the hemostatic sutures were first placed on the lateral side of cervix at 3 and 9 o’clock positions. Afterward, the anterior part of sausage was held by forceps and laterally to the leukoplakia area from the outer part of planned transformation zone. By using number 11 scalpel blade, a circumferential incision was made toward the endocervical canal to excise the cervical tissue as a cone. Finally, hemostasis was maintained by a ball electrode, set at 40 watts on the spray mode.

The cost of one model was less than one euro, and vaginal speculum was not used for maintaining the low cost. One electrosurgical generator and grounding pad were used during the practice session. Additionally, a smoke evacuator produced a comfortable working area. Turkey sausages were preferred to be used as  cervix due to the cost effectiveness of the turkey sausages, the low rate of fatty tissue generated a less smoky working climate^(10)^.

After the workshop, a web-based survey was conducted to measure the effectiveness and clinical relevance of this surgical training model. Participants rated their learning levels for theoretical lessons and surgical techniques. In the end, they assessed their confidence levels for pre-training and post-training conditions. The satisfaction and success scores were rated between 1 (very bad) and 5 (very good). Participants were categorized into two groups and statistical analyses were performed with regard to the previous practice of LEEP and CKC. Age of participants, number of obstetrics and gynecology residents and specialists, theoretical lectures, similarity to the real anatomy, hemostasis, better handling of LEEP loop or scalpel, proper excision of dysplastic area, excision in one piece, unharming the vaginal wall during excision, general educational level of training, as well as pre- and post-course confidence levels were evaluated. Since the article is based on a questionnaire method and besides no patient data is used, there is no need for an ethical approval.

### Statistical Analysis

We performed statistical analyses by using IBM SPSS Statistics 25. Additionally, we presented descriptive statistics as either mean ± standard deviation or median, continuous variables as interquartile range, and categorical variables as the frequency with proportions. We used the Two-Way repeated measure analysis of variance for the statistical analysis of pre-/post-test confidence levels and Mann-Whitney U test for the comparison between the two groups.

## Results

All the 34 participants submitted their responses in the web questionnaire. Most of the participants were residents (29/34, 85.3%) and between 25-30 years of age (21/34, 61.8%) (Table 1). Participants were generally satisfied from the theoretical lectures (median score: 4.0). The training model was found to be highly similar to the real anatomy (median score: 4.0), and learning how to control bleeding for hemostasis was not a complex process (median score: 5) (Table 2).

The educative level of the course on the basic surgical steps of LEEP and CKC procedures was evaluated with regard to the previous practice/expertise of LEEP and CKC. Ten participants (10/34, 29.4%) had previously performed LEEP, whereas 12 participants (12/34, 35.3%) had previously performed CKC. On the contrary, participants in each group had similar learning gains, irrespective of previous expertise (p>0.05) (Table 3).

**Figure 2:**
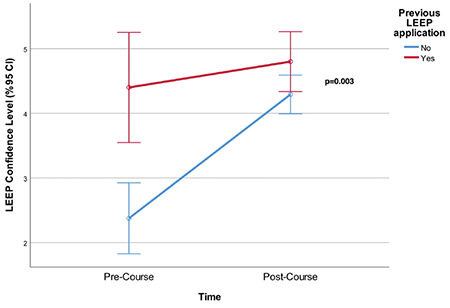
The increase in the mean confidence levels of participants who previously had expertise/practice of Loop Electrosurgical Excision Procedure (LEEP) and participants who did not any expertise/practice of LEEP. The increase was statistically significant for non-practitioners (p=0.003) LEEP: Loop Electrosurgical Excision Procedure, CI: Confidence interval

**Figure 3:**
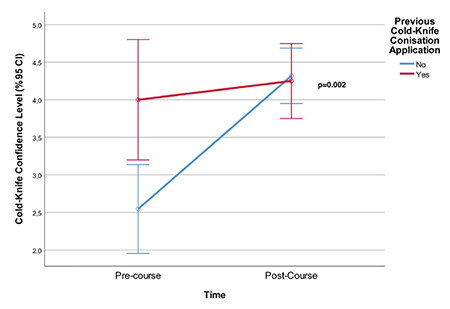
The increase in the mean confidence level of participants who had previous practice/expertise of cold-knife conization (CKC) and participants who did not any expertise of CKC. The increase was statistically significant for non-practitioners (p=0.002) CI: Confidence interval

Moreover, pre-course and post-course mean confidence levels during the conduct of procedures were also analyzed in terms of previous practice of LEEP and CKC. Despite a significantly higher pre-course confidence level for participants who had previously performed LEEP (p<0.001) and CKC (p<0.001) (against the participants who have never performed LEEP or CKC) (Table 4, time variable), the post-course confidence levels were similar in each group among the participants who had either previously performed LEEP or CKC or had not performed LEEP or CKC (p=0.127 and p=0.845, respectively). Additionally, in both groups, participants had increased mean confidence scores, which were statistically significant for participants who have not previously performed the procedure [p=0.003 (Figure 2) p=0.002 (Figure 3) (Table 4, group variable), respectively].

## Discussion

There is a need of post-graduate education and hands-on workshops, especially if the resident is still on the learning stage of a specific type of procedure during the obstetrics and gynecology residency. The key point we found in this research is that a simple and cheap training model on LEEP and CKC procedures will provide satisfactory and successful results in terms of gaining the adequate technical knowledge and skills.

HPV infection is a public health problem, the prevalence of cervical premalignant lesions has increased with time. Many of these patients need a colposcopic evaluation, and LEEP provides an excellent diagnostic and therapeutic option in the management of CIN. Despite similar efficacy and clinical success rates, the CKC procedure is performed in the selected cases because of the relatively higher risk of positive margin status or thermal artifact during LEEP^(1,4)^. Over the last two decades, the creativity-directed post-graduate surgical education toward the simulation projects outside the operating theater/room both for the safety of patient and completion of learning curve. There are few studies targeting the training models that aim to improve the surgical skills during LEEP; however, this study is the first one to assess the CKC technique within a training model^(10,11,12,13)^.

A well-equipped and practical educational system during residency under the supervision of senior surgeons will improve the knowledge and confidence levels during the independent practice of surgical procedures. The more you practice, the more you will gain experience. Additionally, the lack of practice decreases the preparedness of obstetrics and gynecology residents in the post-graduate or fellowship period^(14)^. Overcoming this deficiency and reducing the concerns on the risk of probable complications or an unsuccessful operation will be achieved by attending the hands-on practical courses on training models or cadavers^(11,15)^.

One of the major problems during the simulation of a surgical procedure is the cost of the model. Although the cadaveric workshops are held with a limited number of participants to manage self-practice or dissection under the guidance of a mentor, the course fees are not economical for the residents^(16,17)^. Moreover, the 3D-printed or virtual reality models also provide a good surgical experience with quite more reproducibility; nevertheless, these are neither simple techniques nor cheap models^(18)^. Hefler et al.^(11)^ suggested the use of sausages to mimic the uterine cervix as they resemble the real tissue both in resistance and thickness. Connor et al.^(10)^ recommended the use of sausages with lower fatty content to produce less smoke. Beef or pork meat may also be used, even though it is much better not to prefer the previously frozen or thawed ones^(12)^. During this study the cost of one model was less than one euro, and this LEEP and CKC hands-on training model provided a high yield of reproducibility in a cost effective manner, especially for low-income regions.

Performing a procedure first on a live patient will increase the strain both on the patient and surgeon. The patient will be nervous and stressful as she will be aware of the fact that the resident will be practicing his/her first surgical procedure on her; simultaneously, the resident surgeon will also be anxious. Additionally, one major difficulty of LEEP procedure is the narrow space of vagina, which may cause injuries on the vaginal wall. Operating on a narrow field without adequate experience while the patient is awake will be stressful as well as increase the incidence of complications and decrease the levels of learning. Additionally, before operating on the live surgeries, completing the training steps on the models will improve the learning curve and provide better practice^(13,19)^.

The coordination of hand and eye movements is very important to achieve the best surgical outcomes without harming the nearby tissue, vagina, rectum, or bladder. The excision of the ectocervical tissue in one piece with a correct speed by using the hot wire loop requires adequate practice; otherwise, the excised tissue may be unsuitable for histopathologic evaluation, may not consist the total dysplastic area, or may have a wide thermal artifact^(12)^. This hands-on model aimed to increase the technical ability to coordinate the hand movements; however, performing the procedure without moving the model was the disadvantage detected by us. To overcome this problem, the mentor kept the model stable. If the model is stabilized on the table with a tape or fixed to a specific board, then this will decrease the unintentional movements during the surgical practice. A colposcopic evaluation before LEEP provides an adequate view of the transformation zone, and if there is a doubt of residual lesion after one piece of excision with LEEP loop, then a smaller or rectangular loop will maintain an additional resection. For the first time, a LEEP course was conducted via this study in Turkey, and we mainly focused on the practice of LEEP to improve the skills of gynecologists during the hand movements. From this point, performing an additional resection or endocervical curettage was the limitation of this course.

The widespread use of colposcopy-directed cervical biopsy or LEEP decreased the rates of CKC procedure; nevertheless, it is still a remarkable diagnostic and therapeutic tool in the selected cases. When there is a need of high-volume tissue resection or when the histo-pathologic evaluation has shown critical outcomes such as glandular or microinvasive lesions, CKC is mostly preferred treatment. CKC requires general anesthesia, and bleeding is an important complication^(20)^. Despite many variations in the surgical technique^(21)^, we used the lateral hemostatic sutures at 3 and 9 o’clock positions, and the mentor described the proper borders with regard to the bladder and rectum to totally excise the dysplastic area.

Despite carrying a higher risk for complications due to the relative difficulty of procedure, CKC could successfully be performed by the residents after a well-equipped practical training period; however, the recent literature analyses did not reveal a course targeting to improve the skills for the CKC procedure^(22)^. During the course, the participants also performed the surgical steps of CKC on the sausage, hemostatic sutures, circumferential incision, cone-shaped excision, and cauterization of the cervix.

This study showed a higher degree of pre-course confidence level for a previous expertise of LEEP or CKC; however, the confidence levels were similar for participants who had a previous expertise or those did not at the end of the course. This result showed that participants who did not have any previous expertise had a higher rate of increased confidence levels. On the contrary, the learning gains were similar irrespective of the previous expertise/practice among the LEEP and CKC groups. Ultimately, the course produced a better source of understanding and learning with the purpose of improving technical skills related to LEEP and CKC.

Although the results were successful and promising for improving the surgical technique and skills, drawing a conclusion based on these results can invite misinterpretation.

### Study Limitations

Small number of sample size in the study cohort and subjective grading are the limitations of this research.

## Conclusion

The LEEP and CKC hands-on training model is simple, cheap, and promising to improve the surgical skills and confidence levels before performing the procedure on a real operation setting.

## Figures and Tables

**Table 1:**
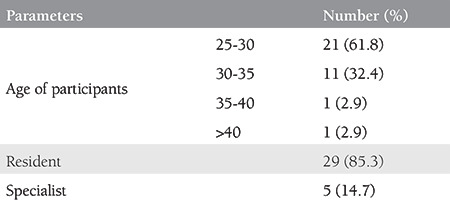
General characteristics of the participants of Loop Electrosurgical Excision Procedure and cold-knife conization hands-on practice course

**Table 2:**
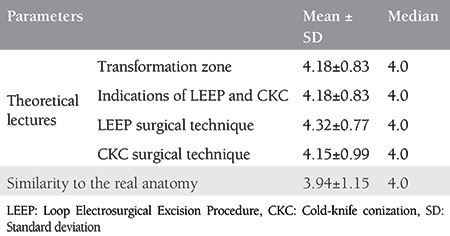
Mean and median scores for theoretical lessons and the model

**Table 3:**
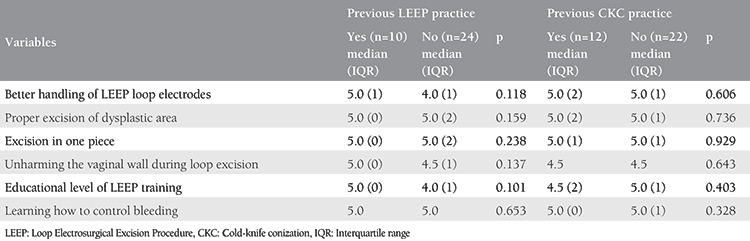
Educative level of the course on the basic surgical steps of Loop Electrosurgical Excision Procedure and cold-knife conization procedures

**Table 4:**
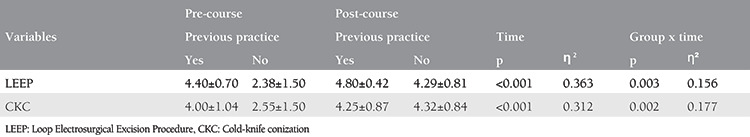
Pre-course and post-course confidence levels regarding the previous practice of Loop Electrosurgical Excision Procedure and cold-knife conization
